# Conserved pigment pathways underpin the dark insectiform floral structures of sexually deceptive *Chiloglottis* (Orchidaceae)

**DOI:** 10.3389/fpls.2022.976283

**Published:** 2022-10-05

**Authors:** Darren C. J. Wong, James Perkins, Rod Peakall

**Affiliations:** Ecology and Evolution, Research School of Biology, The Australian National University, Canberra, ACT, Australia

**Keywords:** *Chiloglottis*, anthocyanin, sexual deception, transcriptome, phylogenomics, Orchidaceae, flavonol glycoside, pollination

## Abstract

Sexually deceptive plants achieve pollination by enticing specific male insects as pollinators using a combination of olfactory, visual, and morphological mimicry. The sexually deceptive orchid genus *Chiloglottis* is comprised of some 30 species with predominantly dull green-red flowers except for the dark insectiform calli/callus structure from the labellum lamina. This unique structure mimics the female of the pollinator and potentially enhances the visibility of the mimic. However, the chemical and genetic basis for the color of these structures remains poorly understood across the genus. The goal of this study was to investigate the flower color biochemistry and patterns of gene expression across the anthocyanin and flavonol glycoside biosynthetic pathway within the calli structures across the three distinct clades of *Chiloglottis* (Formicifera, Reflexa, and Valida) using chemical and transcriptome analysis. Our phylogenomic analysis confirmed the close sister relationship between the Reflexa/Formicifera clades and reaffirms the basal position of the Valida clade. Additionally, the biochemical basis of the dark calli/callus structures is conserved across the genus. Nonetheless, the proportion of methoxylated anthocyanin and flavonol glycoside derivatives and the mean gene expression levels appear to differentiate the Reflexa and Formicifera clades from the Valida clade. In future studies, it will be of interest to tease apart the role of phylogeny, environment, pollinators, and other factors as potential drivers of the observed biochemistry and gene expression differences. It will also be important to characterize the function of candidate genes such as *DFR*, *LDOX,* and *FLS* in this fascinating case of flower color mimicry.

## Introduction

Sexually deceptive plants achieve pollination by enticing specific male insects as pollinators using a combination of olfactory, visual, and morphological mimicry of female insects. Olfactory mimicry is the best-studied trait, but visual mimicry is perhaps the most striking aspect of sexually deceptive flowers to humans. Most sexually deceptive flowers are predominantly dull red and green, aside from one or several prominent dark insect-like (insectiform) floral structures on the labellum. These structures, which can appear as stalked calli (de Jager and Peakall, 2016), fine “hairs” (Peakall, 1990; Vereecken et al., 2012; Vignolini et al., 2012; Cohen et al., 2021), and wart-like bumps (Gaskett and Herberstein, 2010; Gaskett et al., 2017), are thought to be visual and tactile cues that enhance the insect mimicry.

**Figure 1:**
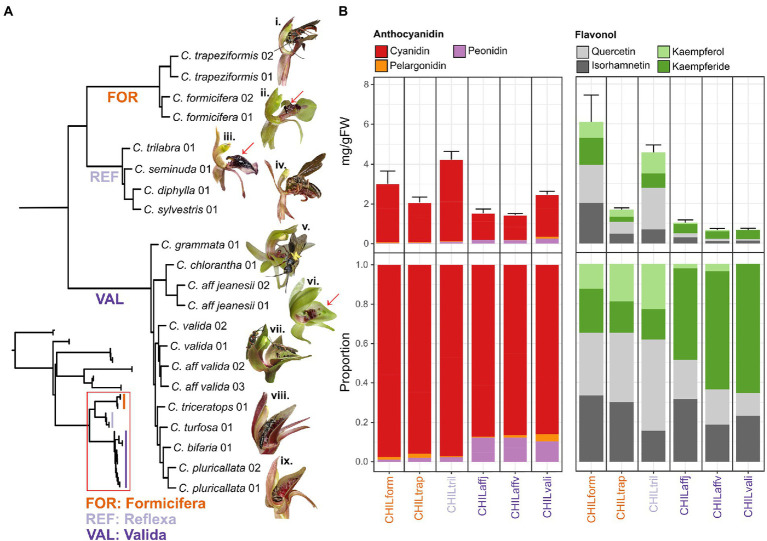
Phylogenetic patterns of anthocyanin and flavonol glycosides in a cross-section of Chiloglottis orchids. **(A)** The full (inset, top left) and close up (red boxed) ASTRAL species tree phylogeny of *Chiloglottis* (red boxed) with and without representatives from *Arthrochilus*, *Caleana*, *Drakaea*, *Paracaleana*, and *Spiculaea* genera as outgroup is illustrated, respectively. Representative flowers from the FOR (**i.** *C. trapeziformis*, **ii.** *C. formicifera*), REF (**iii.** *C. trilabra*, **iv.** *C. diphylla*), and VAL (**v.** *C. chlorantha*, **vi.** *C. aff. jeanesii*, **vii.** *C. aff. valida*, **viii.** *C. turfosa*, **ix.** *C. bifaria*) clades are shown. Red arrows point to the dark, three-dimensional insectiform calli/callus structure on the labellum lamina of representative FOR, REF, and VAL clades. Floral traits in each clade typically range as follows—FOR: flowers 6–8 mm wide × 12–16 mm long; labellum 6–7 mm wide × 8–10 mm long; length to width ratio 1.3–1.4 ×, REF: flowers 4–9 mm wide × 10–34 mm long; labellum 5–8 mm wide × 9–11 mm long; length to width ratio 1.5–1.9 ×, and VAL: flowers 15–30 mm wide × 18–30 mm long; labellum 9–14 mm wide × 10–17 mm long; length to width ratio close to 1. **(B)** Bar graphs showing total anthocyanins and flavonol glycoside co-pigment content grouped by aglycone (average ± s.e.) and their corresponding proportion in the calli/callus of naturally opened flowers. See Supplementary Figure S3 for full ASTRAL species tree phylogeny and Supplementary Figures S4, S5 for detailed flowering time, flower size, and labellum size traits.

The sexually deceptive orchid genus *Chiloglottis* contains approximately 30 species occurring predominantly in eastern Australia and Tasmania, but with one species extending to New Zealand and sub-Antarctic islands (Jones, 2021). *Chiloglottis* species typically have dark maroon to black calli/callus which contrast with their otherwise green or dull red labellum lamina (Figure 1). To the human eye, the calli/callus resemble an insect perched on the flower. These structures serve dual roles: (1) as the primary source of chiloglottones, a class of 2,5-dialkylcyclohexan-1,3-dione natural products that are sexually attractive to the thynnine wasp pollinators of the group (Schiestl et al., 2003; Franke et al., 2009; Peakall et al., 2010; Wong et al., 2017, 2018, 2019), and (2) as a visual and tactile mimic of the female (de Jager and Peakall, 2016). Other floral traits have also been shown to be relevant to pollinator attraction and pollination. These include the amount (Schiestl, 2004) and composition of the semiochemicals used for pollinator attraction (Peakall et al., 2010), flower height (Peakall and Handel, 1993), and labellum size (de Jager and Peakall, 2016, 2019).

Our study builds on this large body of prior work, and in particular on recent work on the chemical composition and genetic regulation of the temporal and spatial color patterns underpinning the visual mimicry in *Chiloglottis trapeziformis* (Wong et al., 2022). Targeted metabolite profiling in this species revealed that the localized distribution of abundant cyanidin-based anthocyanins and diverse flavonol glycoside co-pigments give the black callus structure its distinct dark color. Developmental stage and tissue-specific differential expression of genes encoding dihydroflavonol reductase, leucoanthocyanidin dioxygenase, flavonol synthase, and flavonoid O-methyltransferase that controls specific branch point into respective anthocyanin and flavonol glycoside biosynthesis (and their methylated derivatives) may also assist in maintaining the color from the earliest bud stage through to the mature flower. These findings highlight the multi-gene and regulatory complexity of the floral color adaptations involved in securing pollination by sexual deception (Wong et al., 2022).

In this perspective article, phylogenetically-informed sampling across the three distinct clades of *Chiloglottis,* each with characteristic phenological and morphological traits, were performed to assess whether there are any phylogenetic differences in flower color biochemistry and patterns of gene expression within the calli. We first leveraged a new phylogenomic approach to confirm if phylogenetic relationships within and among the major clades continue to hold with many additional loci. Next, targeted metabolite profiling and transcriptomes analysis of representative *Chiloglottis* were performed to determine whether floral color pathways that are largely responsible for the distinct coloration of “insectiform” calli is conserved (see Supplementary Figure S1 for a general framework). Our results confirmed that the biochemistry and biosynthetic machinery is broadly conserved across three major clades of the genus. However, they also revealed some phylogenetic differences. We conclude with a discussion and suggestions for further research to aid in teasing apart the role of phylogeny, environment, pollinators, and other factors that may drive these observed biochemistry and gene expression shifts.

### Phylogenomic relationships of *Chiloglottis*

Molecular phylogenetic studies of *Chiloglottis* based on limited nuclear and plastid sequences (Mant et al., 2002, 2005; Peakall et al., 2010; Miller and Clements, 2014) have consistently indicated that there are three major clades: Reflexa (REF), Formicifera (FOR) and Valida (VAL). Here we performed a targeted sequence assembly based on a customized multitiered sequence capture strategy (see Supplementary Methods) of 14 *Chiloglottis* species (18 samples obtained in this study, see Supplementary Table S1 for details) and 17 samples from an earlier study encompassing three *Chiloglottis* (3 samples) and 12 non-*Chiloglottis* (14 samples) species belonging to the Drakaeinae with representatives from *Arthrochilus*, *Caleana*, *Drakaea*, *Paracaleana*, and *Spiculaea* (Peakall et al., 2021).

Although our analysis encompasses some 5.8 million bp of sequence and spans both exonic and non-exonic (e.g., potential homologous untranslated regions, introns, and other off-target nuclear, chloroplast, and mitochondrial DNA) regions (Supplementary Figure S2), the shortcut coalescent ASTRAL phylogeny was broadly congruent with earlier studies (Figure 1A). Notably, the close sister relationship between the REF/FOR clades, and the basal position of the VAL clade was reconfirmed (Peakall et al., 2010). Very short branches and high levels of gene tree discordance continue to be observed within the clades (Figure 1A; Supplementary Figure S3 for details). These results point to widespread incomplete lineage sorting and rapid radiation within clades, a characteristic also noted as a feature of other sexually deceptive orchid genera (Bateman et al., 2018, 2021; Peakall et al., 2021; Wong and Peakall, 2022).

### Distinguishable labellum and insectiform calli traits across the *Chiloglottis* clades

Drawing on a recent flora of Australian orchids (Jones, 2021), we have mapped the flowering time, flower size, and labellum size traits of our study species across the phylogeny (Supplementary Figures S4, S5). Generally, members of each clade share a characteristic flowering time: REF in late summer to autumn (December–May), FOR in late winter to spring (August–November), and VAL in late winter to summer (range August–February). The sister REF/FOR clades share a common floral morphology with small single flowers [REF: 5–7 (*w*) × 17–21 (*l*) mm, FOR: 6–8 (*w*) × 12–16 (*l*) mm average] borne on taller peduncles (40–120 mm high). All flowers have a slim labellum for the proximal first third before expanding to a broadly triangular and spathulate (diamond) shape toward the tips, with their other petals reflexed against the ovary. The labella bear clusters of black calli that appear insectiform to human eyes. These highly clustered calli occupy 30%–80% of the total labellum area. By contrast, the single larger flowers (25–30 × 13–23 mm average) of the VAL clade are held closer to the ground (15–75 mm high), while the flowers have a larger and wider chordate-shaped labellum, with larger petals that are either held outward or incurved upwards. Furthermore, while the flowers also bear dark calli, these tend to cover a smaller proportion (10–40%) of the total labellum area, and generally appear much less insectiform to human eyes (see also Figure 1 legend for floral sizes by clade).

### Floral color chemistry of *Chiloglottis* calli

Targeted metabolite profiling was performed to determine whether the floral color chemistry is conserved across the phylogeny. For each of the six *Chiloglottis* species surveyed, the calli and/or callus structures were first dissected from labellum lamina for subsequent anthocyanin/flavonol extraction and analysis by UHPLC–MS/MS (Supplementary Table S2) and LC-DAD-MS (Supplementary Figure S6). Five anthocyanins and 16 flavonol glycosides were detected with total anthocyanin content (expressed as mg g^−1^ FW) ranging between 1.42–4.21 in the calli (Figure 1B). The composition of anthocyanidins was broadly consistent across the three clades with cyanidin-based anthocyanins as the dominant component (85%–99%) followed by peonidin- (1%–12%) and pelargonidin- (0%–4%) based anthocyanins as minor constituents. However, the proportion of peonidin-based anthocyanins were consistently higher in the VAL clade (10%–12%) compared to members of the FOR and REF (1%–3%) clades.

Total flavonol glycoside content (expressed as mg g^−1^ FW per flower) ranged between 0.64–6.07 in the calli (Figure 1B), with *C. formicifera* and *C. trilabra* showing markedly higher total flavonol glycoside content in their calli/callus compared to *C. trapeziformis*, *C. aff. valida*, *C. aff. jeanesii*, and *C. valida*. Four main types of flavonol glycosides were found, with the proportions of quercetin (Q)-, isorhamnetin- (IR), kaempferol- (K), and kaempferide- (Kde) based flavonol glycosides ranging between 11%–46%, 16%–44%, 0%–23%, and 16%–65%, respectively. Interestingly, Q and its methylated derivative, IR together often made up 62%–65% of total flavonol glycoside content in FOR and REF groups, whereas Kde alone make up 46%–65% of total flavonol glycoside across the VAL group. While variable across species, in general, the ratios of total anthocyanins to flavonol glycosides in the calli, were higher in VAL group members (1.5–3.8 to 1) than in FOR and REF group members (0.9–1.2 to 1).

Collectively, these findings indicate a phylogenetic pattern of compositional differences in anthocyanin, and especially flavonol glycoside pigment chemistry of the calli between VAL and REF/FOR flowers with higher proportions of methoxylated derivatives (peonidin, kaempferide, and isorhamnetin) appears to be a characteristic of the VAL clade. Here, we observed that the chemical basis of these black “insectiform” structures also shares surprising parallels observed in the European alpine food-rewarding orchid, *Gymnadenia rhellicani* (Kellenberger et al., 2019) where its high anthocyanin content, composed of cyanidin 3-O-glucoside and cyanidin 3-O-(6″-malonyl-glucoside) in the petal and labellum (similar composition observed across *Chiloglottis*), provides some flower morphs its black color. Beyond orchids, uniformly black flowers (Markham et al., 2004; Thill et al., 2012) or dark markings/spots in petals (Vignolini et al., 2015; van der Kooi and Stavenga, 2019) and unusual black 3D floral structures (Thomas et al., 2009) is also due to an oversaturation of anthocyanins, often in the presence of co-pigments.

### Pigment biosynthetic pathways of *Chiloglottis* calli

**Figure 2:**
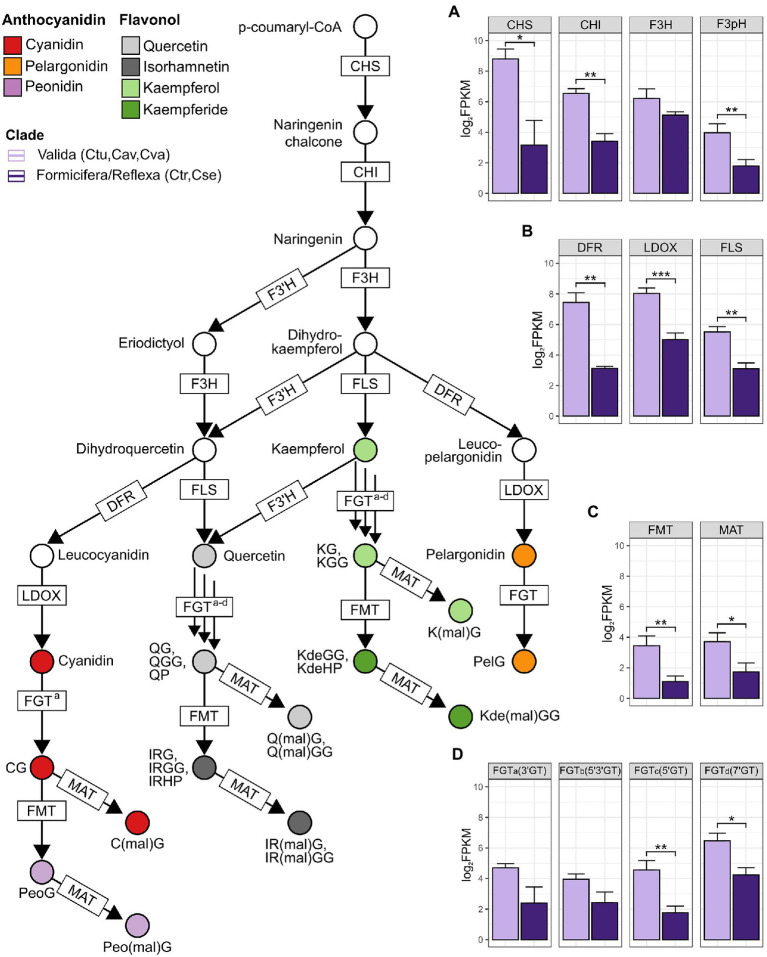
Phylogenetic patterns of anthocyanin/flavonol glycoside-related pathway expression of mature flower calli in a cross-section of *Chiloglottis* orchids. Boxplots from **(A–D)** depict the general patterns of genes in respective pathway steps across the three clades—REF (*Chiloglottis seminuda*), FOR (*Chiloglottis trapeziformis*), and VAL (*Chiloglottis turfosa*, *Chiloglottis aff. valida*, *Chiloglottis valida*). Asterisks indicate significant differences in the total gene expression between clade comparisons at *p* < 0.05 (*), *p* < 0.01 (**), and *p* < 0.001 (***) based on ANOVA and Tukey’s HSD test. See Supplementary Table S3 for full gene names and predicted function of corresponding gene symbols.

Floral transcriptomes of species belonging to FOR (*C. trapeziformis*), REF (*C. seminuda*), and VAL (*C. turfosa*, *C. aff. valida*, and *C. valida*) clades were also investigated. The same sets of candidate genes encoding shared flavonoid and dedicated anthocyanin and flavonol glycoside pathway enzymes in *C. trapeziformis* were also identified in the representative REF and VAL species (Figure 2; Supplementary Table S3). Additionally, genes involved in the modification (e.g., glycosylation, methylation, and acylation) of anthocyanidins and flavonols at one or more positions were also identified. Generally, the diversity of anthocyanins and flavonol glycosides detected in the callus is in accord with the expression of candidate genes downstream of F3’H (two cyanidin and two peonidin anthocyanins, five Q and five IR flavonol glycosides) and those that do not depend on F3’H activity such as one pelargonidin anthocyanin, three K, and three Kde flavonol glycosides (Figure 1; Supplementary Figure S6; Supplementary Table S2).

The patterns of anthocyanin and flavonol glycoside-related pathway gene expression were also investigated. Mean levels of gene expression were compared across two groups: the three species representing the VAL clade (*C. turfosa*, *C. aff. valida,* and *C. valida*), and the pooled single representatives each of the REF (*C. seminuda*) and FOR (*C. trapeziformis*) group. The outcomes of this analysis are summarized in Figure 2, which maps the patterns of gene expression onto the anthocyanin/flavonol glycoside-related pathway. The mean levels of gene expression were frequently significantly higher in the VAL group than the REF/FOR group. For example, this pattern was found at the shared flavonoid (*CHS*, *CHI*, and *F3H*), dedicated anthocyanin (*DFR* and *LDOX*), and flavonol glycoside (*FLS*) biosynthesis genes. Significantly higher mean expression levels were also detected for some downstream modification genes (e.g., *FMT, MAT*, *5′GT*, and *7′GT*; Figure 2; Supplementary Figure S7) in the VAL group. These findings indicate a strong phylogenetic pattern of gene expression difference in the anthocyanin and flavonol glycoside pathways of the calli at the open flower stage when the calli were dissected in this study.

### Environmental or pollinator-driven differences in biochemistry and gene expression?

*Chiloglottis* orchids are embedded within a well-resolved subtribe (Diurideae: Drakaeinae) in which all genera are sexually deceptive barring a few self-pollinating cases (Weston et al., 2014; Peakall et al., 2021). Because of this, floral adaptations associated with insect mimicry have likely been finely tuned by a long evolutionary process. Many studies have shown that differences in seasons, day length, light quality, and temperature additively affect the flavonoid composition of plant tissues (Jaakola and Hohtola, 2010). For example, shading or light exclusion significantly increased peonidin anthocyanins compared to exposed skins of berries (i.e., grapevine and bilberries) while cyanidin anthocyanins were generally unaffected (Downey et al., 2004; Zoratti et al., 2014). Some co-occurring *Chiloglottis* species flower at different times of the year (Jones, 2021), meaning that environmental conditions are likely to vary even among sympatric species (Supplementary Figure S4). Indeed, there are distinctly different flowering times for each of the three clades, with FOR being mainly spring flowering, REF being mainly autumn flowering, and VAL being mainly spring and summer flowering (Jones, 2021). Consequently, it is possible that seasonal differences affect the developmental conditions of the flowers and may partially account for the anthocyanin/flavonol glycoside compositional differences observed between the clades.

Interestingly, strong experimental evidence for pollinator-mediated selection on the floral traits that distinguish the VAL and FOR/REF clades is indicated by studies of one pair of *Chiloglottis* species. *C. trapeziformis* (FOR) and *C. valida* (VAL) use the same semiochemical, chiloglottone 1, to attract their own phylogenetically distinct wasp pollinators, *Neozeleboria cryptoides* and *N. monticola,* respectively. Key floral trait differences between the two orchids are strongly correlated with behavioral differences in approach, landing position, and orientation during attempted copulation by the two pollinators (de Jager and Peakall, 2016). However, in this study, the largest anthocyanin/flavonol glucoside compositional differences were found between these two species. Therefore, it seems unlikely that seasonal differences and distinct floral morphological features can fully explain the observed differences. Could these composition differences instead reflect pollinator preference? Although direct experiments on wasp pollinator color preferences are lacking, spectral reflectance measurements provide some clues. When projected into hymenopteran vision space, the measurements indicate the calli/callus colors are very similar. In both orchids these structures are predicted to be perceived as achromatic to the male pollinator(s), matching the achromaticity of the females (de Jager and Peakall, 2016). Thus, it also seems unlikely that pollinator color preference could have driven the anthocyanin/flavonol glycoside composition differences observed between the VAL and REF/FOR clades.

## Conclusion and future directions

Selection for flower color has been predicted to be stronger for plants with specialized pollinator interactions compared to those with multiple pollinators involving many diverse taxonomic groups (Trunschke et al., 2021). As expected the biochemical and biosynthetic basis of the dark calli/callus structures of the labellum is conserved across the genus (Wong et al., 2022). However, subtle shifts in anthocyanin and flavonol glucoside biochemistry such as a higher proportion of methoxylated derivatives, and differences in gene expression were evident between the VAL clade and the sister REF/FOR clades. In order to better understand the basis for these observed expression differences, a first step is to confirm that peak anthocyanin/flavonol glucoside and corresponding gene expression levels are consistently shifted to earlier developmental stages in REF/FOR clades compared to VAL clades. One promising candidate pair of study species to first test this hypothesis would be *C. trapeziformis* and *C. valida,* where there is occasional co-flowering and pollinator sharing.

Further investigation into the tissue-specific flower pigmentation patterns (e.g., calli/callus vs. labellum lamina) across the clade also warrants further investigation. In *C. trapeziformis*, increased accumulation of anthocyanins in the callus relative to the labellum lamina at flowering (Wong et al., 2022) results in strong within-flower chromatic/achromatic contrast, potentially aiding detectability by pollinators (de Jager and Peakall, 2016). However, for many other species from the FOR/REF clades, the black calli occupy nearly the entire labellum lamina (Supplementary Figure S5) leaving little to no room for within-flower contrast. The sexually deceptive *Drakaea livida* shares this characteristic, and in this case, the entire labellum is contrasted with the background substrate (Gaskett et al., 2017). Similarly, the dominance of the calli in FOR and REF clade species may promote contrast of the whole labellum with the background rather than within-flower contrast which is common in the VAL clade.

Innovative field experiments will be required to tease apart the importance of the color from the structure of the calli. Working with rare flower color variants may prove particularly informative for determining the importance of color to pollination success. Alternatively, working with 3D printed flowers with varying calli/callus colors at varying flower developmental stages is another promising avenue, and has been effective in *Dracula* orchids (Policha et al., 2016). In all cases, it will remain essential to hold the chemistry of pollinator attraction constant. Here the use of synthetic chiloglottones can be effectively used, as already demonstrated by the multiple studies that have investigated the role of chemistry in *Chiloglottis* pollinator attraction (e.g., Schiestl et al., 2003; Peakall et al., 2010). In future studies, it will also be of interest to tease apart the key signals (developmental and/or environmental), the function of candidate genes (e.g., *DFR*, *LDOX*, *FLS*, and *FMT*), and to more fully evaluate the role of pollinators in this fascinating case of flower color mimicry.

## Data availability statement

Publicly available datasets were analyzed in this study. This data can be found at: NCBI PRJNA661963, PRJNA486025, and PRJNA390683.

## Author contributions

DW and JP performed the experiments. RP and DW secured the funding, designed the study, and coordinated the experiments and data analysis. DW wrote the article with assistance from RP and JP. All authors contributed to the article and approved the submitted version.

## Funding

This work was supported by Australian Research Council Projects (DE190100249 to DW and DP1094453 and DP150102762 to RP) and an Australian Government Research Training Program to JP.

## Conflict of interest

The authors declare that the research was conducted in the absence of any commercial or financial relationships that could be construed as a potential conflict of interest.

## Publisher’s note

All claims expressed in this article are solely those of the authors and do not necessarily represent those of their affiliated organizations, or those of the publisher, the editors and the reviewers. Any product that may be evaluated in this article, or claim that may be made by its manufacturer, is not guaranteed or endorsed by the publisher.
