# Supplementary material for: Conserved pigment pathways underpin the dark insectiform floral structures of sexually deceptive Chiloglottis (Orchidaceae)
Source: Front Plant Sci. 2022 Oct 5;13:976283. doi: 10.3389/fpls.2022.976283 (PMC9581149; doi:10.3389/fpls.2022.976283)
Supplement: Supplementary file 2 [file Data_Sheet_2.PDF]

SUPPLEMENTARY METHODS

Conserved pigment pathways underpin the dark insectiform floral structures of sexually deceptive *Chiloglottis* (Orchidaceae)

Darren C.J. Wong[1†], James Perkins[1], Rod Peakall[1]

[1]Ecology and Evolution, Research School of Biology, The Australian National University, Acton ACT 2601, Australia

[†]**Correspondence:**
Darren CJ Wong
darren.wong@anu.edu.au; wongdcj@gmail.com
+61 2 6125 9892

Sample collection

Leaves and single naturally opened flowers of *Chiloglottis* plants were sourced from wild populations within the Australian National Botanic Gardens in ACT (Australia) and Kosciuszko National Park in NSW (Australia) for targeted sequence capture and anthocyanin analysis (See **Table S1** for details). Samples were immediately snap frozen in liquid nitrogen at the point of collection and stored at -80°C. For targeted sequence capture experiments, 18 samples from 14 *Chiloglottis* species were obtained for this study. Samples included several replicates from one (*C. trapeziformis*) or more sites (*C. formicifera*, *C. pluricallata*, *C. aff. valida*, *C. valida*, and *C. aff. jeanesii*) as well as species that potentially share morphological and genetic affinities with related members (*C. aff. valida*, *C. aff. jeanesii*). For anthocyanin analysis, floral tissues of six *Chiloglottis* taxa (*Chiloglottis formicifera*, *C. trapeziformis*, *C. trilabra*, *C. aff. jeansii*, *C. aff. valida*, *C. valida*) were carefully dissected to separate the calli (*cal*) from the labellum lamina.

Targeted sequence capture and assembly

A customized multitiered sequence capture strategy targeting multiple phylogenetic depth across Diurideae, i.e. at the tribe (Sets 1 and 2) and subtribe level (e.g. other subtribes with uncertain placement within the Diurideae – Sets 3, within Drakaeinae – Sets 4, and within Caladeniinae – Sets 5) were used in this study (Peakall et al. 2021). Detailed steps involving DNA extraction, library preparation, and Illumina sequencing were performed as previously described (Peakall et al. 2021). Briefly, DNA extractions were performed with the Qiagen DNeasy Plant mini kit (Cat. No. 69106) according to the manufactures protocol. DNA quality was verified by gel electrophoresis and subsequently quantified using the Quant-iT ds DNA High-Sensitivity Assay Kit (Invitrogen Q33120, Thermo Fisher Scientific, USA) on the Tecan microplate reader (Tecan Trading AG, Switzerland). When possible, 1mg of DNA per sample in 110 ul of water was used for library construction. Before library construction, DNA from each sample was sheared to an average of 500 bp in size with Biorupter (Diagenode Diagnostics, Belgium) and checked for shearing efficiency with microchip electrophoresis using MultiNA (Shimadzu Corporation, Kyoto, Japan). Size selection was then performed to retain fragments between 200 and 700 bp using Sera-Mag bead clean-up (GE Healthcare Life Sciences, Australia).

Sample barcoding and genomic library construction was then performed according Rohland and Reich (2012) and Meyer and Kircher (2010), respectively. Exon capture hybridization and washing was performed following the manufacturer's protocol (SeqCap EZ Developer Library; Roche NimbleGen) followed by target enrichment and indexing via PCR for 12 cycles. The final library was quantified and quality checked using Agilent Bioanalyser (Agilent Technologies, Santa Clara, California, USA). Sequencing was performed on an Illumina NextSeq500 (150 bp paired-end) at the Biomolecular Resource Facility, The Australian National University. For each sample, initial post-sequencing processing and quality control (i.e. adaptor removal, trimming, and quality filtering) was performed using Trimmomatic v0.36 (Bolger et al. 2014) before sequence assembly using Trinity (Grabherr et al. 2011) with previously described parameters (Peakall et al. 2021).

Homologous *k*-mer block discovery and phylogenetic analysis

Following targeted sequence capture assembly into contigs, homologous *k*-mer blocks (i.e. sets of syntenic, nearly exact matching, single-copy *k*-mers extended by an ungapped region flanking

each *k*-mer) within assembled contigs that are shared between samples were identified with Hakmer (Sanderson et al. 2017). All parameters were default except for *k*-mer length, $k = 25$; maximum number of allowed mismatch, $q = 2$; minimum taxonomic coverage, $N_{min} = 12$, width of flanking sequence, $w = 25$; and pooling of neighbouring *k*-mer blocks with two or more individual blocks present on the same contig option enabled. Homologous *k*-mer blocks, both single and pooled were concatenated as supermatrices for subsequent species tree inference.

Individual locus (gene) trees with branch support of respective homologous *k*-mer blocks were first inferred using IQ-TREE v2 (Minh et al. 2020b) using the best-fit partitioning scheme and substitution model as determined by ModelFinder (Kalyaanamoorthy et al. 2017), followed by tree search and branch support assessment using ultrafast bootstrap with 1000 replicates (UFBoot; Hoang et al. 2018). Shortcut coalescent species tree was inferred using ASTRAL-MP (Yin et al. 2019), a multi-parallel implementation of ASTRAL-III (Zhang et al. 2018) with default parameters. Gene trees of homologous *k*-mer blocks (from IQ-TREE) with extremely low support (i.e. UFBoot ≤ 20) were contracted and the resulting multifurcating trees were used as input. ASTRAL tree branch support was estimated using local posterior probabilities (localPP). Gene concordance factor (gCF) was calculated using individual locus (gene) tree against the ASTRAL inferred species tree using the *-gcf* option (Minh et al. 2020a). Visualisation and comparative analysis of phylogenetic trees were achieved using iTOL v4 (Letunic and Bork 2019).

Ultra high-performance liquid chromatography mass spectrophotometric (UHPLC-MS/MS) analysis

Targeted metabolite analysis was performed as previously described using ultra high-performance liquid chromatography mass spectrophotometric (UHPLC-MS/MS) analysis (Wong et al. 2022). Briefly, samples were homogenised and extracted in 400 µL of solvent composed of 70:30:1 methanol:water:acetic acid with agitation at 4°C for 24 hours followed by centrifugation at 13,000 rpm for 10 minutes. The supernatant was filtered with 0.2 um PTFE SINGLE StEP filter vials (Thomson) and analysed using a Thermo Q Extractive Plus UPLC-Orbitrap Mass Spectrometer (Thermo Scientific, Waltham, MA, USA). Chromatographic separation of samples and standards (5 µL injection volumes) were performed on an Agilent reversed-phase Zorbax Eclipse XDB-C18 column (2.1 × 50 mm, 1.8 µm particles) held at 40°C. All solvents were HPLC grade or higher. The mobile phases used were: water with 0.1% formic acid (solvent A), and methanol with 0.1% formic acid (solvent B). Samples and standards were eluted with a constant flow rate of 400 µL min-1, with a 25-minute gradient program as follows: 0-1.5 min, 6% B; 1.5-2 min, 6-10% B; 2-14 min, 10-60% B; 14-15 min, 60-90% B; 15-19 min, 90% B; 19-20 min, 6% B; 20-25 min, 6% B. Eluted compounds were introduced to the MS via a HESI-II probe (Thermo Scientific, Waltham, MA, USA). To better distinguish anthocyanins from flavonol glycosides, a separate LC-MS instrument fitted with a diode array detector (DAD) operating at 520 nm and at 365 nm was used to analyse representative samples using the same chromatographic parameters.

For putative identification and relative quantification of anthocyanins and flavonol glycosides, the HESI was operated in the positive mode. Mass spectra were acquired using full MS and data-dependant MS/MS acquisition (DDA) modes at a scan range of 100 to 1500 m/z. Representative samples of both tissue types were also analysed in negative ionisation mode. Data acquisition and analysis was achieved using Thermo Scientific XCALIBUR 4.0 and Thermo Scientific FreeStyle

software, respectively. Anthocyanins were distinguished from flavonol glycosides based on (1) the 4 decimal place accurate masses of molecular ions in full MS spectra in both ionisation modes, (2) absorbance spectra at 520 and 365 nm, (3) comparisons of fragmentation patterns in the MS/MS spectra in both ionisation modes with online repositories such as RIKEN tandem mass spectral database (Sawada et al. 2012) and MassBank of North America (https://mona.fiehnlab.ucdavis.edu/), and (4) the absence or presence of the diagnostic $[M-2H+H_2O]^-$ ion in the negative ionisation analyses. Putative anthocyanins and flavonol glycosides were quantified by integration of the molecular ion peak in full MS spectra and calculated from linear calibration curves of cyanidin 3-O-glucoside chloride (Sigma-Aldrich, USA) and 7-hydroxycoumarin (Sigma-Aldrich, USA), respectively.

De novo transcriptome assembly

Paired-end reads (PE) of *C. seminuda*, *C. turfosa*, *C. aff. valida*, and *C. valida* were obtained from respective NCBI Bioproject/SRA accessions. Adaptor clipping, sliding-window trimming, quality and length (l = 40) filtering of the raw PE reads were achieved with *fastp* v0.20.0 (Chen et al. 2018) using default settings unless otherwise specified. Transcriptome assembly were performed using *Trinity* v2.11.0 (Haas et al. 2013) with default settings except a minimum contig length threshold of 300 and *k*-mer size of 31. Protein-coding prediction was performed with TransDecoder v5.5.0 (http://transdecoder.github.io) and EvidentialGene tr2aacds4 pipeline (Don Gilbert 2013) using default settings. For TransDecoder, the recommended homology searches against the UniProt Reference Clusters (i.e. UniRef90) and protein families (i.e. Pfam) databases were performed using DIAMOND (Buchfink et al. 2014) and HMMER (Mistry et al. 2013), respectively and incorporated into the TransDecoder pipeline to maximize the prediction sensitivity.

Transcriptome analysis and annotation

Filtered PE reads were aligned against the re-assembled transcriptomes of *C. seminuda*, *C. turfosa*, *C. valida*, and *C. aff. valida* individually with bowtie2 (Langmead and Salzberg 2012) using the local read alignment mode (*--local*). Count tables for each species were generated with FeatureCounts (Liao et al. 2014) using default parameters except for the *-B* (i.e. both ends must be aligned) and *-C* (i.e. exclude chimeric fragments) option enabled. Transcript expression were expressed as Fragments Per Kilobase of transcript per Million mapped reads (FPKM). Annotation of MapMan functional category, Pfam domain, and putative homologs were achieved using Mercator (Lohse et al. 2014) and TransDecoder (http://transdecoder.github.io). For *C. trapeziformis*, the transcriptome dataset and associated information were retrieved from Wong et al. (2022).

REFERENCES

Bolger AM, Lohse M, Usadel B (2014) Trimmomatic: A flexible trimmer for Illumina sequence data. Bioinformatics 30:2114–2120 . https://doi.org/10.1093/bioinformatics/btu170

Buchfink B, Xie C, Huson DH (2014) Fast and sensitive protein alignment using DIAMOND. Nat Methods 12:59–60 . https://doi.org/10.1038/nmeth.3176

Chen S, Zhou Y, Chen Y, Gu J (2018) Fastp: An ultra-fast all-in-one FASTQ preprocessor.

Bioinformatics 34:i884–i890 . https://doi.org/10.1093/bioinformatics/bty560

Don Gilbert (2013) EvidentialGene: tr2aacds, mRNA transcript assembly software. http://arthropods.eugenes.org/EvidentialGene/trassembly.html

Grabherr MG, Haas BJ, Yassour M, Levin JZ, Thompson D a, Amit I, Adiconis X, Fan L, Raychowdhury R, Zeng Q, Chen Z, Mauceli E, Hacohen N, Gnirke A, Rhind N, di Palma F, Birren BW, Nusbaum C, Lindblad-Toh K, Friedman N, Regev A (2011) Full-length transcriptome assembly from RNA-Seq data without a reference genome. Nat Biotechnol 29:644–652 . https://doi.org/10.1038/nbt.1883

Haas BJ, Papanicolaou A, Yassour M, Grabherr M, Blood PD, Bowden J, Couger MB, Eccles D, Li B, Lieber M, Macmanes MD, Ott M, Orvis J, Pochet N, Strozzi F, Weeks N, Westerman R, William T, Dewey CN, Henschel R, Leduc RD, Friedman N, Regev A (2013) De novo transcript sequence reconstruction from RNA-seq using the Trinity platform for reference generation and analysis. Nat Protoc 8:1494–1512 . https://doi.org/10.1038/nprot.2013.084

Hoang DT, Chernomor O, Von Haeseler A, Minh BQ, Vinh LS (2018) UFBoot2: Improving the ultrafast bootstrap approximation. Mol Biol Evol 35:518–522 . https://doi.org/10.1093/molbev/msx281

Kalyaanamoorthy S, Minh BQ, Wong TKF, Von Haeseler A, Jermiin LS (2017) ModelFinder: Fast model selection for accurate phylogenetic estimates. Nat Methods 14:587–589 . https://doi.org/10.1038/nmeth.4285

Langmead B, Salzberg SL (2012) Fast gapped-read alignment with Bowtie 2. Nat Methods 9:357–359 . https://doi.org/10.1038/nmeth.1923

Letunic I, Bork P (2019) Interactive Tree of Life (iTOL) v4: Recent updates and new developments. Nucleic Acids Res 47:256–259 . https://doi.org/10.1093/nar/gkz239

Liao Y, Smyth GK, Shi W (2014) FeatureCounts: An efficient general purpose program for assigning sequence reads to genomic features. Bioinformatics 30:923–930 . https://doi.org/10.1093/bioinformatics/btt656

Lohse M, Nagel A, Herter T, May P, Schroda M, Zrenner R, Tohge T, Fernie AR, Stitt M, Usadel B (2014) Mercator: A fast and simple web server for genome scale functional annotation of plant sequence data. Plant, Cell Environ 37:1250–1258 . https://doi.org/10.1111/pce.12231

Meyer M, Kircher M (2010) Illumina sequencing library preparation for highly multiplexed target capture and sequencing. Cold Spring Harb Protoc 5:1–11 . https://doi.org/10.1101/pdb.prot5448

Minh BQ, Hahn MW, Lanfear R (2020a) New Methods to Calculate Concordance Factors for Phylogenomic Datasets. Mol Biol Evol 37:2727–2733 . https://doi.org/10.1093/molbev/msaa106

Minh BQ, Schmidt HA, Chernomor O, Schrempf D, Woodhams MD, Von Haeseler A, Lanfear R, Teeling E (2020b) IQ-TREE 2: New models and efficient methods for phylogenetic inference in the genomic era. Mol Biol Evol 37:1530–1534 . https://doi.org/10.1093/molbev/msaa015

Mistry J, Finn RD, Eddy SR, Bateman A, Punta M (2013) Challenges in homology search: HMMER3 and convergent evolution of coiled-coil regions. Nucleic Acids Res 41:e121 . https://doi.org/10.1093/nar/gkt263

Peakall R, Wong DCJ, Phillips RD, Ruibal M, Eyles R, Rodriguez-Delgado C, Linde CC (2021) A multitiered sequence capture strategy spanning broad evolutionary scales: Application for phylogenetic and phylogeographic studies of orchids. Mol Ecol Resour 21:1118–1140 . https://doi.org/10.1111/1755-0998.13327

Rohland N, Reich D (2012) Cost-effective, high-throughput DNA sequencing libraries for multiplexed target capture. Genome Res 22:939–946 . https://doi.org/10.1101/gr.128124.111

Sanderson MJ, Nicolae M, McMahon MM (2017) Homology-aware phylogenomics at gigabase scales. Syst Biol 66:590–603 . https://doi.org/10.1093/sysbio/syw104

Sawada Y, Nakabayashi R, Yamada Y, Suzuki M, Sato M, Sakata A, Akiyama K, Sakurai T, Matsuda F, Aoki T, Hirai MY, Saito K (2012) RIKEN tandem mass spectral database (ReSpect) for phytochemicals: A plant-specific MS/MS-based data resource and database. Phytochemistry 82:38–45 . https://doi.org/10.1016/j.phytochem.2012.07.007

Wong DCJ, Perkins J, Peakall R (2022) Anthocyanin and flavonol glycoside metabolic pathways underpin floral color mimicry and contrast in a sexually deceptive orchid. Front Plant Sci 13:860997 . https://doi.org/10.3389/fpls.2022.860997

Yin J, Zhang C, Mirarab S (2019) ASTRAL-MP: Scaling ASTRAL to very large datasets using randomization and parallelization. Bioinformatics 35:3961–3969 . https://doi.org/10.1093/bioinformatics/btz211

Zhang C, Rabiee M, Sayyari E, Mirarab S (2018) ASTRAL-III: Polynomial time species tree reconstruction from partially resolved gene trees. BMC Bioinformatics 19:153 . https://doi.org/10.1186/s12859-018-2129-y
