# Supplementary material for: Conserved pigment pathways underpin the dark insectiform floral structures of sexually deceptive Chiloglottis (Orchidaceae)
Source: Front Plant Sci. 2022 Oct 5;13:976283. doi: 10.3389/fpls.2022.976283 (PMC9581149; doi:10.3389/fpls.2022.976283)
Supplement: Supplementary file 3 [file Data_Sheet_3.pdf]

SUPPLEMENTARY FIGURES AND TABLES

Conserved pigment pathways underpin the dark insectiform floral structures of sexually deceptive *Chiloglottis* (Orchidaceae)

Darren C.J. Wong[1†], James Perkins[1], Rod Peakall[1]

[1]Ecology and Evolution, Research School of Biology, The Australian National University, Acton ACT 2601, Australia

[†]Correspondence:
Darren CJ Wong
darren.wong@anu.edu.au; wongdcj@gmail.com
+61 2 6125 9892

FIGURE S1. A general framework to understand the biochemical basis of the black 'insectiform' floral structure across *Chiloglottis* employed in this study. To assess whether there are any phylogenetic differences in flower color biochemistry and patterns of gene expression within the calli, phylogenetically-informed sampling across *Chiloglottis* is crucial. We first leveraged a new phylogenomic approach to confirm whether phylogenetic relationships within and among the major clades continue to hold with many additional loci (analysis/steps in blue). Mapping the floral traits of our target species onto the phylogeny further revealed distinguishable labellum and insectiform calli traits across the *Chiloglottis* clades (green). Next, targeted metabolite profiling (analysis/steps in orange) and transcriptomes analysis (analysis/steps in purple) of representative *Chiloglottis* were performed to determine whether floral color pathways are responsible for the distinct coloration of 'insectiform' calli are largely conserved. † target sequence capture data obtained in this study; * target sequence capture data obtained from Peakall et al. (2021); ‡ floral traits data obtained from Jones (2021); § transcriptome data obtained from Peakall et al. (2021), Wong et al. (2019), and Wong et al. (2022). Please refer to **Supplementary Methods** section for further details on the analysis/steps highlighted here.

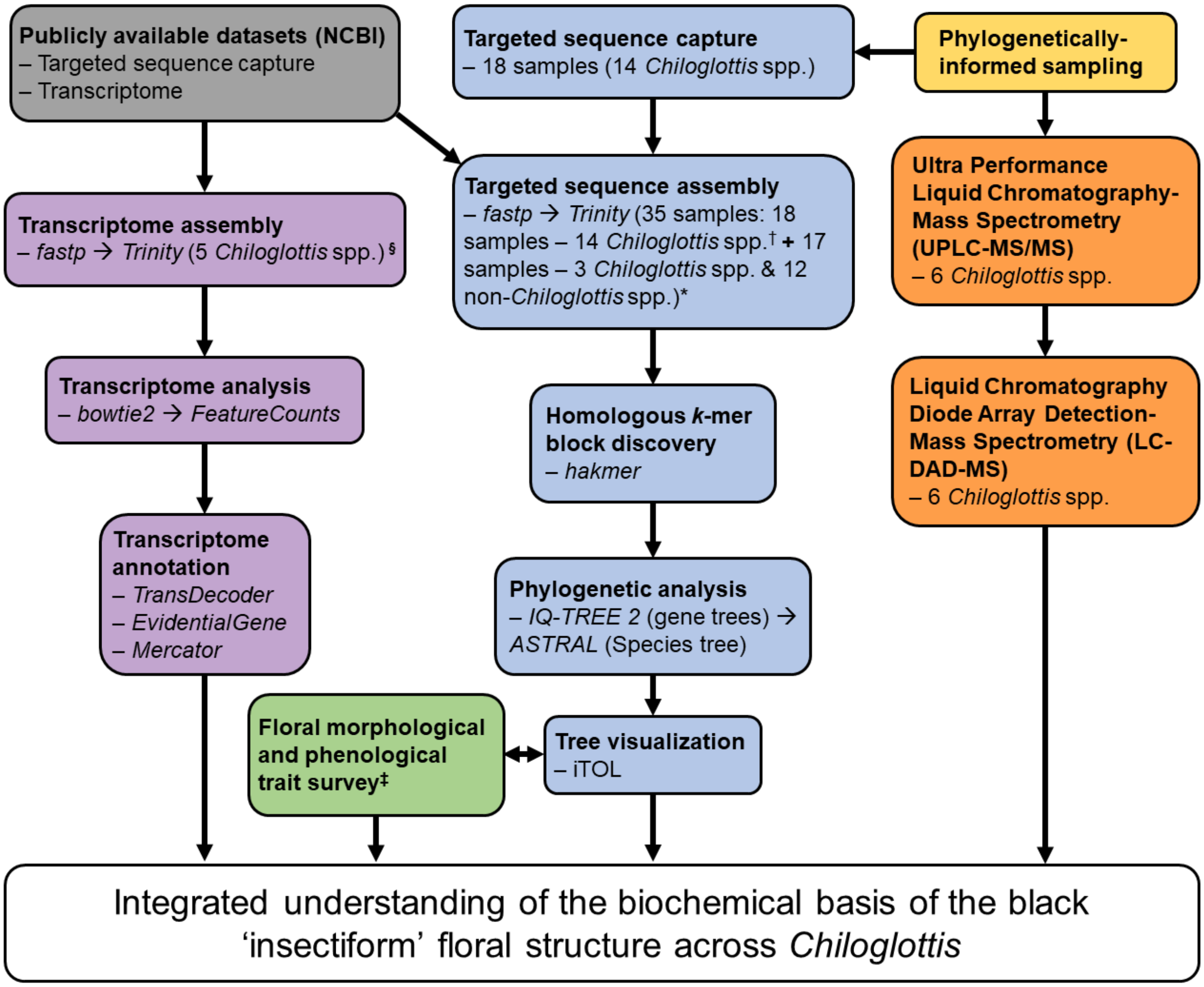

FIGURE S2. Composition of homologous *k*-mer blocks used for phylogenetic inference of the Drakaeinae (35 samples: 21 samples corresponding to 17 *Chiloglottis* species and 14 samples encompassing representatives from *Arthrochilus*, *Caleana*, *Drakaea*, *Paracaleana*, and *Spiculaea* genera. A total of 78,665 homologous *k*-mer blocks were present in at least 12 (of 35) samples. Prior to phylogenetic analysis, neighbouring *k*-mer blocks (i.e. two or more individual blocks) present on the same contig, *M* were first pooled. Together, a final set of 38,558 *k*-mer blocks (total alignment of 5,899,875 bp with 773,198 parsimony-informative sites) encompassing 18,835 *M* pooled (derived from 58,568 individual blocks) and 19,723 singleton (i.e. unpooled) blocks were used shortcut coalescent (Zhang et al. 2018) species tree inference. (**A**) 78,095 (99.3%) *k*-mer blocks have their corresponding contigs in respective species aligned to all five target sequence sets. (**B**) The number of *k*-mer blocks recovered for *Chiloglottis* were higher compared to non-*Chiloglottis* species (median of 49,000 vs. 26,000, respectively). Large proportion of the 78,095 shared *k*-mer blocks mapped to target loci of Sets 4 (35 – 45%), followed by Sets 3 (~ 25%), Sets 5 (~ 10 – 15%), Sets 1 (~ 8%), and Sets 2 (~ 10 – 17%). (**C**) Each shared *k*-mer block can be further categorized into 3 main classes – mapping to CDS only (using the target sequence sets as reference), CDS + flank, flank only – to reflect their underlying exon and/or non-exonic composition. The latter may include homologous untranslated regions, introns, and other off-target nuclear, chloroplast and mitochondrial DNA sequences, among others. Interestingly, there were more (~ 2x) shared *k*-mer blocks that flanked the CDS entirely (i.e. LEFT or RIGHT flanking 5p3pITR) rather mapping exclusively within them (i.e. 5pCDS_3pCDS) for *Chiloglottis*. Compared to non-*Chiloglottis* samples, these numbers are roughly comparable. Shared *k*-mer blocks whose parts mapped within CDS but contain flanking regions were also observed but in smaller proportions (~25%) with a median of 31 and 30nt overhang in either 5p or 3p direction from mapped CDS (data not shown). (**D**) The median number and percentage of the total loci recovered in *Chiloglottis* and non-*Chiloglottis* species were 1047 (99%) and 994 (94%) sequences, respectively. As expected, a high proportion of the target loci belonged to Sets 4. High target loci recovery were observed for Sets 1, 2, and 3 (75 – 94%) but poorly for Sets 5 (~ 50%).

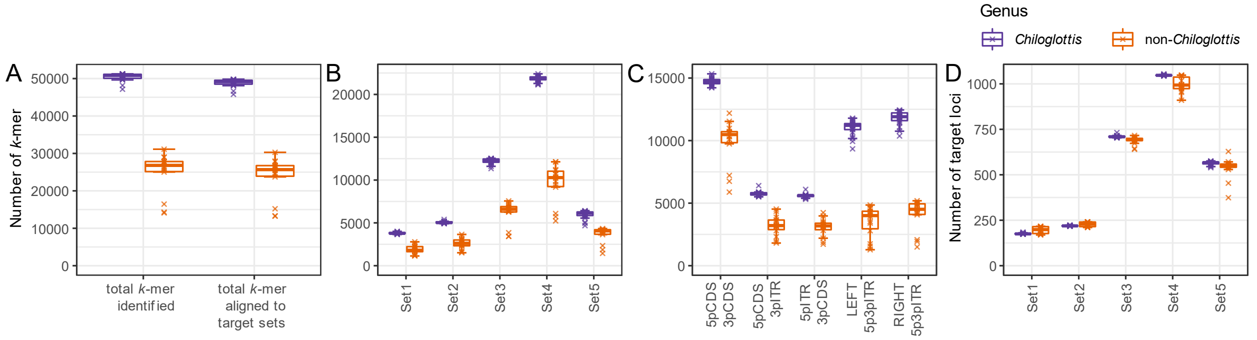

FIGURE S3. The estimated ASTRAL species tree phylogeny of *Chiloglottis* (with representatives from *Arthrochilus*, *Caleana*, *Drakaea*, *Paracaleana*, and *Spiculaea* genera as outgroup). The phylogeny is based on the quartet analysis of the gene trees estimated by IQ-TREE 2 for 38,558 *k*-mer blocks (total alignment of 5,899,875 bp with 773,198 parsimony-informative sites). All but two branches received local posterior probabilities < 1 (indicated by *). Branch colors indicate gene concordance factor (gCF) values – the proportion of gene trees which decisively support the presented bifurcation. Scale bar indicates coalescent units. Inset indicates the relationships of gCF against branch length in coalescent units across the depicted phylogeny. Briefly, the Valida group (nine spp.) is placed sister to a clade formed by Formicifera (two spp.) and Reflexa (four spp.) members. Within Valida, *C. grammata* is sister to all other species in this clade. This is followed by a clade containing *C. chlorantha* and *C. aff. jeanesii* that is sister to another clade characterised by very short branches and low gene concordance factors (6 spp.: *C. aff. valida*, *C. valida*, *C. triceratops*, *C. turfosa*, *C. sp* (*bifaria*), and *C. pluricallata*). Only two nodes concerning the relationship of *C. valida* and *C. aff. valida* received localPP of 0.9 and 0.92, respectively. Additionally, all species with multiple samples from collection site(s) that span large (200–600 km apart, e.g. *C. aff. jeanesii* and *C. valida*) or finer (<100 km, e.g. *C. aff. valida*, *C. pluricallata*) geographic scales were paired together. Within Reflexa, *C. trilabra* is basal and is placed sister to *C. seminuda*, followed by a clade containing *C. sylvestris* and *C. diphylla*. Within Formicifera, *C. trapeziformis* and *C. formicifera* is sister with one another and replicate samples also paired accordingly. See **Supplementary Data 1** for further details on the homologous *k*-mer block files, corresponding sequence alignment, gene trees, and species tree.

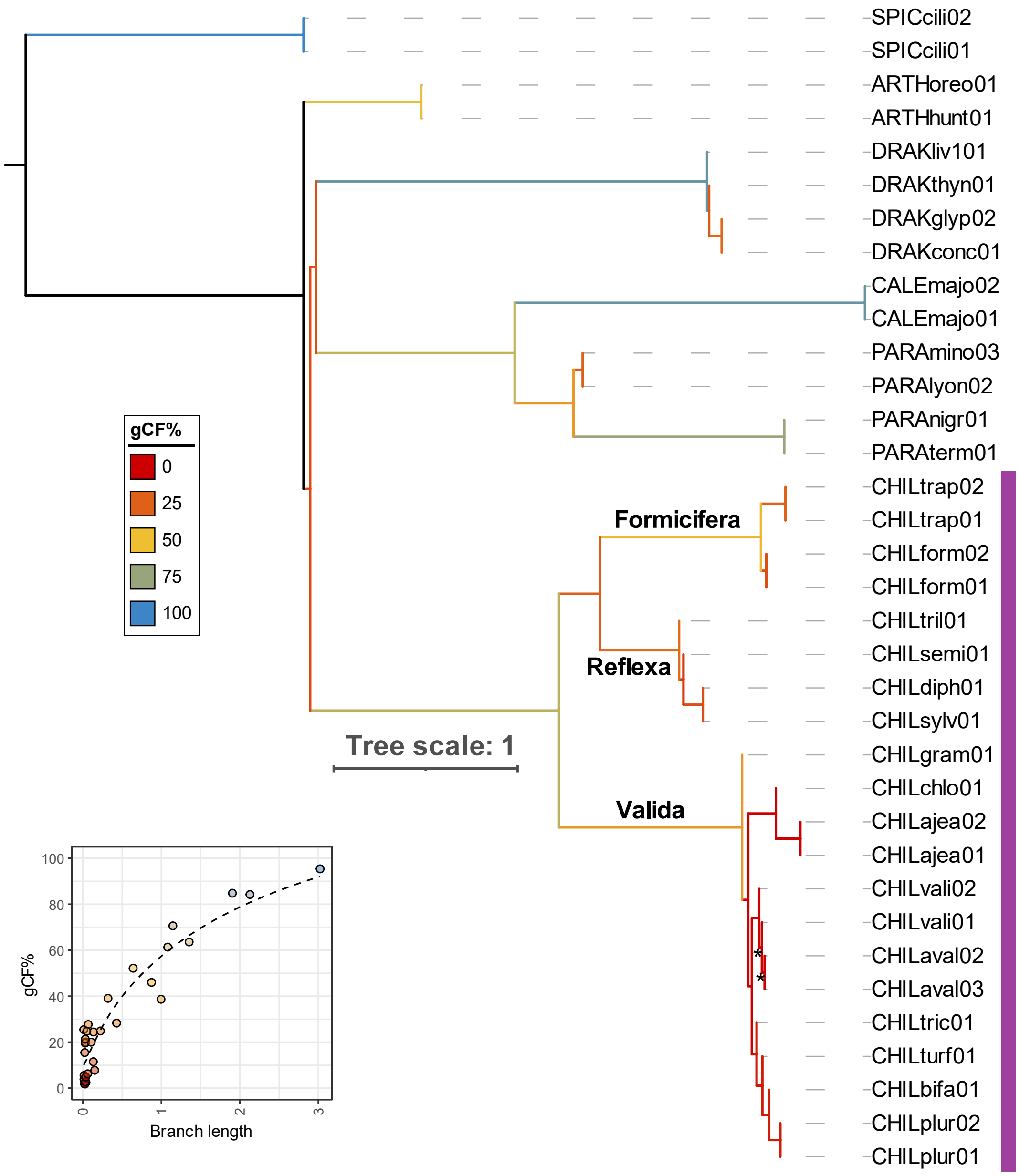

SPICcili02
SPICcili01
ARTHoreo01
ARTHhunt01
DRAKliv101
DRAKthyn01
DRAKglyp02
DRAKconc01
CALEmajo02
CALEmajo01
PARAmino03
PARAlyon02
PARAnigr01
PARAterm01
CHILtrap02
CHILtrap01
CHILform02
CHILform01
CHILtril01
CHILsemi01
CHILdiph01
CHILsylv01
CHILgram01
CHILchlo01
CHILajea02
CHILajea01
CHILvali02
CHILvali01
CHILaval02
CHILaval03
CHILtric01
CHILturf01
CHILbifa01
CHILplur02
CHILplur01
Formicifera
Reflexa
Valida
Tree scale: 1
gCF%
0
25
50
75
100
gCF%
Branch length
0
20
40
60
80
100
0
1
2
3

FIGURE S4. Key floral phenological and morphological traits between major *Chiloglottis* clade representatives and outgroups. L, length; W, width.

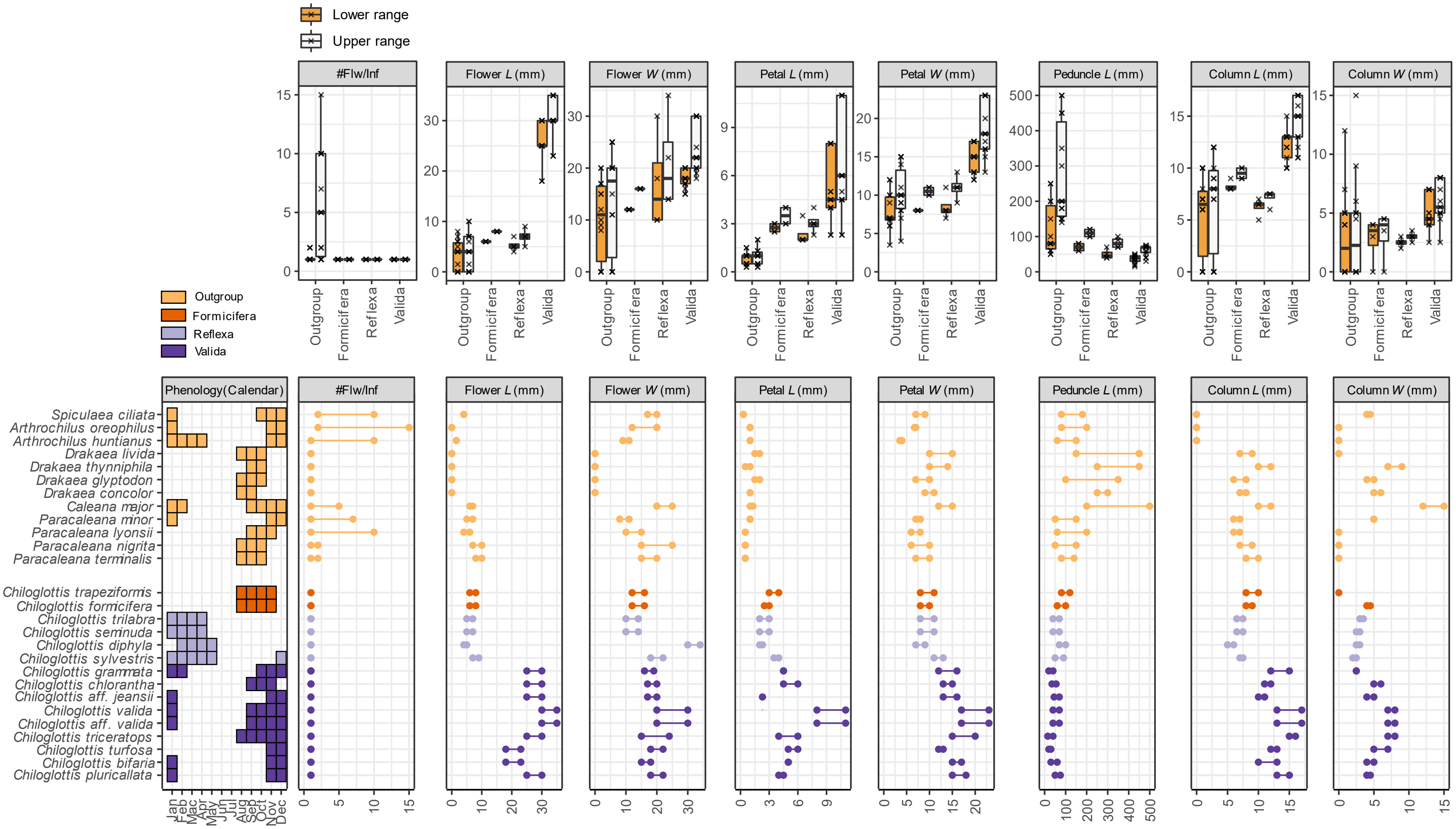

FIGURE S5. Flower labellum morphological traits between major *Chiloglottis* clade representatives and outgroups. L, length; W, width; Lab L:W ratio, the labellum length divided by width; % of Cal cov on Lab, the proportion of the labellum that is covered by calli.

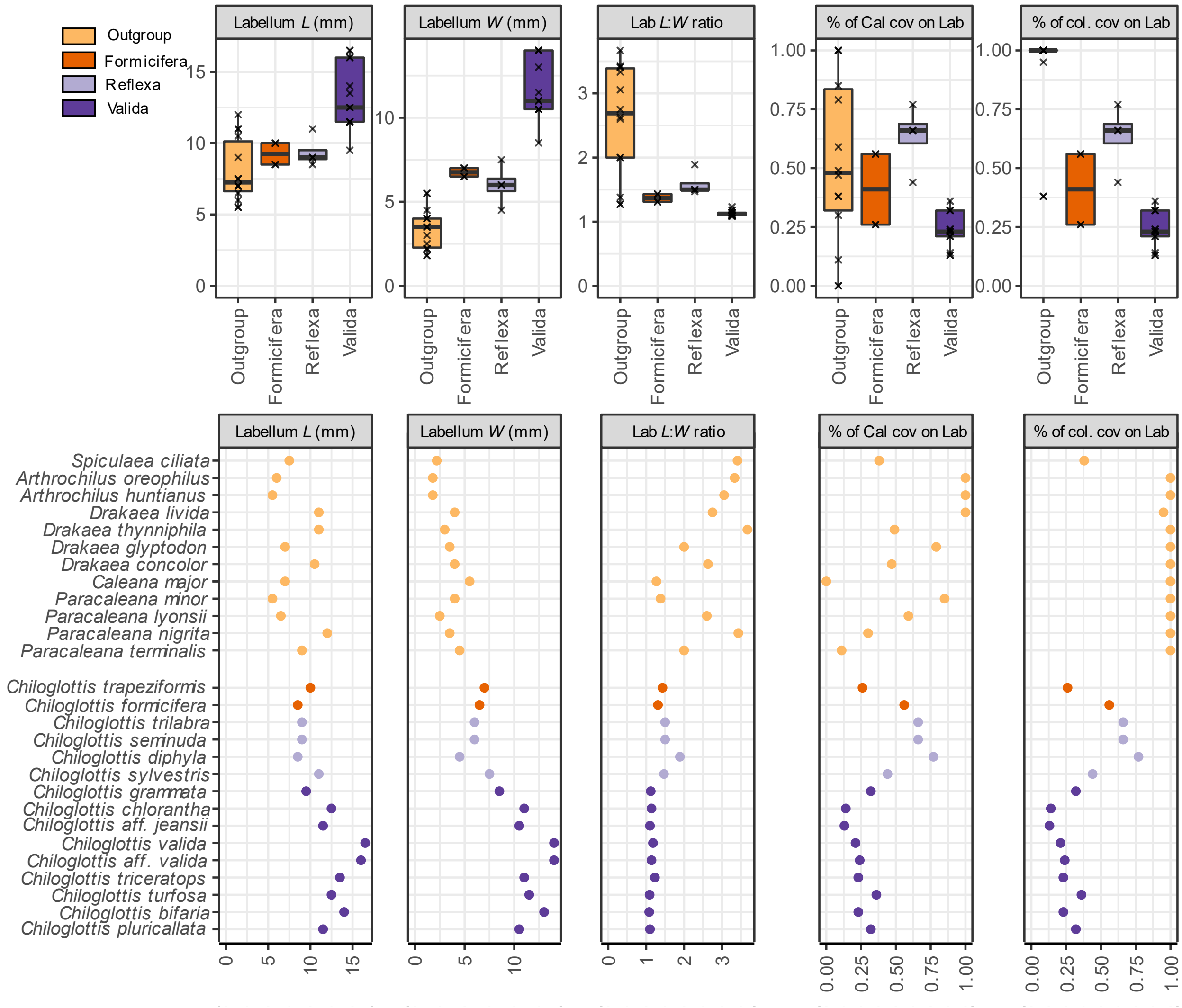

FIGURE S6. High-performance liquid chromatography-diode array detection (HPLC-DAD) spectra of anthocyanins and flavonols in a representative callus extract in a cross-section of mature *Chiloglottis* flowers. Absorbance spectra at (A) 520nm and (B) 365nm are shown. Putative anthocyanins and flavonol glycosides are indicated. ^ indicates flavonol glycosides unique to the Valida group. See **Supplementary Table S2** for additional details obtained from the ultra high-performance liquid chromatography-tandem mass spectrometry runs for individual species.

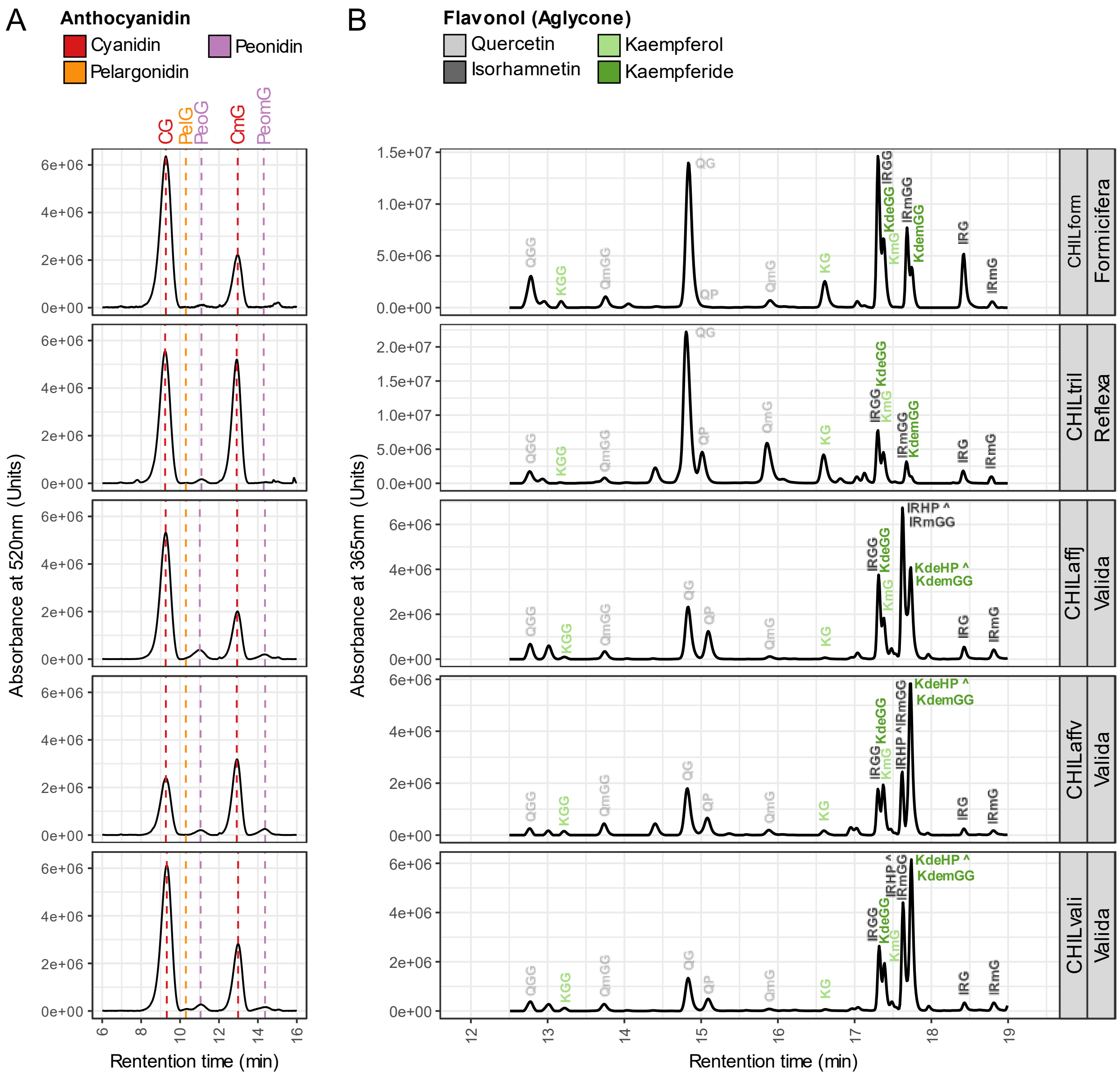

FIGURE S7. Flower calli anthocyanin and flavonol metabolism in a cross-section of *Chiloglottis* flowers. Heatmap in spectral colors depicts the log_2-transformed normalized expression values (log_2 FPKM+1) of related pathway genes in the calli of five *Chiloglottis* species. Circles are colored according to their aglycone and corresponding labels indicate putative anthocyanins and flavonols identified in a cross-section of *Chiloglottis* flowers. See **Supplementary Table S2** for additional details obtained from the ultra high-performance liquid chromatography-tandem mass spectrometry runs and **Supplementary Table S3** for the full gene name and predicted function of corresponding gene symbols, and **Supplementary Data 2** for additional functional annotation.

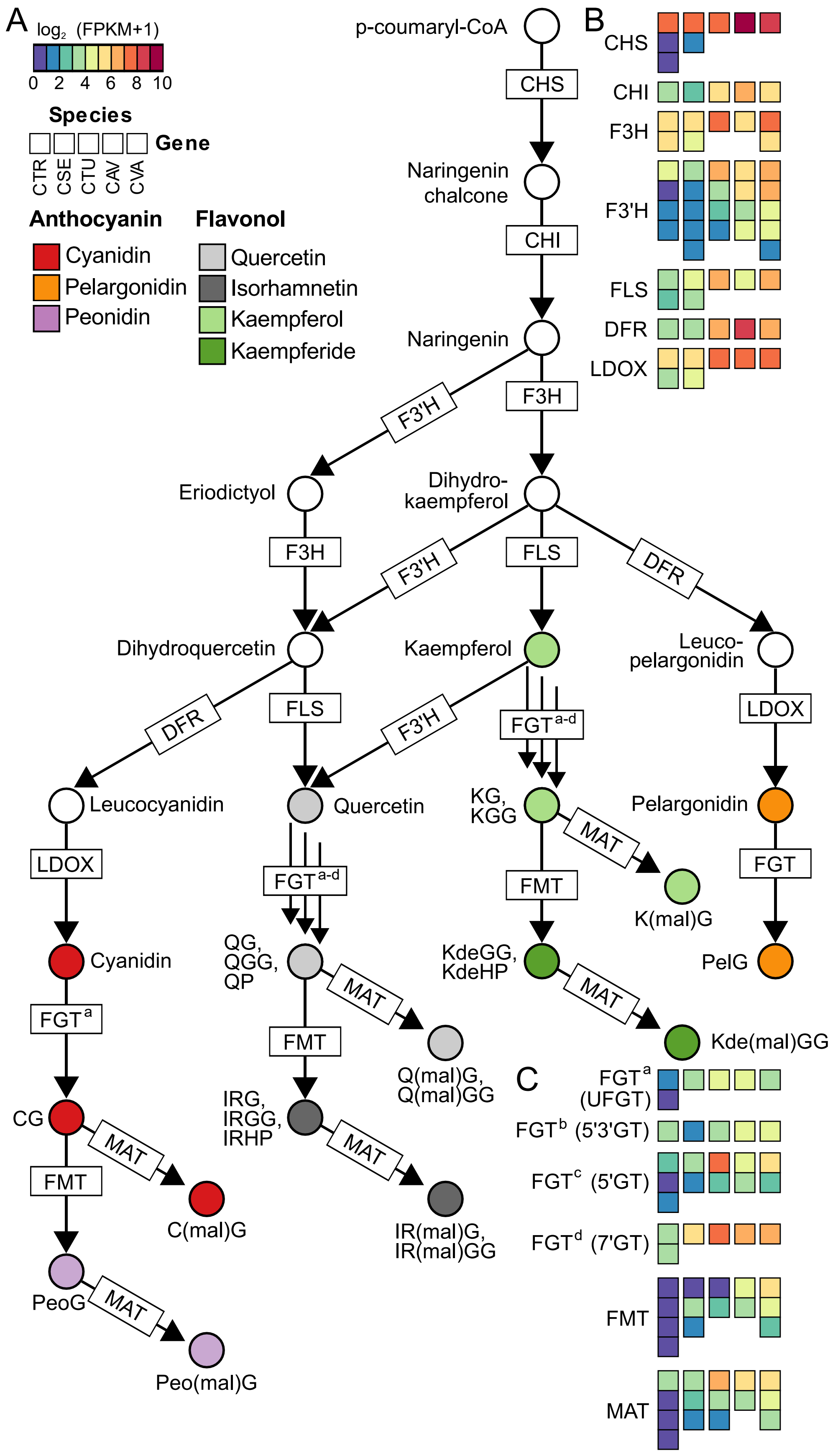

A
log2 (FPKM+1)
0 2 4 6 8 10
Species
Gene
CTR CSE CTU CAV CVA
Anthocyanin
Cyanidin
Pelargonidin
Peonidin
Flavonol
Quercetin
Isorhamnetin
Kaempferol
Kaempferide
p-coumaryl-CoA
CHS
Naringenin chalcone
CHI
Naringenin
F3'H
F3H
Eriodictyol
Dihydro-kaempferol
F3H
F3'H
FLS
DFR
Dihydroquercetin
Kaempferol
Leuco-pelargonidin
DFR
FLS
F3'H
FGTa-d
LDOX
Leucocyanidin
Quercetin
KG, KGG
MAT
Pelargonidin
LDOX
FGTa-d
FMT
K(mal)G
FGT
Cyanidin
QG, QGG, QP
KdeGG, KdeHP
PelG
FGTa
MAT
MAT
FMT
Q(mal)G, Q(mal)GG
Kde(mal)GG
CG
IRG, IRGG, IRHP
MAT
MAT
FMT
C(mal)G
IR(mal)G, IR(mal)GG
PeoG
MAT
Peo(mal)G
B
CHS
CHI
F3H
F3'H
FLS
DFR
LDOX
C
FGTa (UFGT)
FGTb (5'3'GT)
FGTc (5'GT)
FGTd (7'GT)
FMT
MAT

TABLE S1. Details of species used in this study. Species code, source, species designation, location, source information, and analysis purpose are shown. State: ACT, Australian Capital Territory; NSW, New South Wales; TAS, Tasmania; VIC, Victoria. Source: C1, cultivated CSIRO Living Collection ACT; W=wild. Study type: E, Exome; A, Anthocyanin

Code	Source	Species	State	Locality	Source	Study
SPICcili01	Peakall et al. 2021	*Spiculaea ciliata*	WA	Boulder Rock	W	E
SPICcili02	Peakall et al. 2021	*Spiculaea ciliata*	WA	Boulder Rock	W	E
ARTHoreo01	Peakall et al. 2021	*Arthrochilus oreophilus*	QLD	Barron Gorge NP	W	E
ARTHhunt01	Peakall et al. 2021	*Arthrochilus huntianus*	NSW	Kosciuszko NP	W	E
DRAKliv101	Peakall et al. 2021	*Drakaea livida*	WA	Margaret River	W	E
DRAKthyn01	Peakall et al. 2021	*Drakaea thynniphila*	WA	Carey Brook	W	E
DRAKglyp02	Peakall et al. 2021	*Drakaea glyptodon*	WA	Mount Roe NP	W	E
DRAKconc01	Peakall et al. 2021	*Drakaea concolor*	WA	Northhampton	W	E
CALEmajo01	Peakall et al. 2021	*Caleana major*	NSW	Oallen	W	E
CALEmajo02	Peakall et al. 2021	*Caleana major*	NSW	Oallen	W	E
PARAmino03	Peakall et al. 2021	*Paracaleana minor*	ACT	Black Mt NR	W	E
PARAlyon02	Peakall et al. 2021	*Paracaleana lyonsii*	WA	Kalbarri NP	W	E
PARAnigr01	Peakall et al. 2021	*Paracaleana nigrita*	WA	Quinninup	W	E
PARAterm01	Peakall et al. 2021	*Paracaleana terminalis*	WA	Northhampton	W	E
CHILajea01	this study	*Chiloglottis aff jeanesii*	NSW	Tallaganda State Forest	W	E
CHILajea02	this study	*Chiloglottis aff jeanesii*	NSW	Kanagara Boyd NP	W	E
CHILajeaF	this study	*Chiloglottis aff jeanesii*	NSW	Tallaganda State Forest	W	A
CHILaval02	this study	*Chiloglottis aff valida*	VIC	Nolan	W	E
CHILaval03	this study	*Chiloglottis aff valida*	VIC	Hepburn Regional Park	W	E
CHILavalF	this study	*Chiloglottis aff valida*	NSW	Kosciuszko NP	W	A
CHILbifa01	this study	*Chiloglottis bifaria*	NSW	Barrington Tops NP	W	E
CHILchlo01	this study	*Chiloglottis chlorantha*	NSW	Budderoo NP	W	E
CHILdiph01	this study	*Chiloglottis diphylla*	NSW	CSIRO glasshouse	C1	E
CHILform01	this study	*Chiloglottis formicifera*	ACT	CSIRO glasshouse	C1	E
CHILform02	this study	*Chiloglottis formicifera*	NSW	Nattai NP	W	E
CHILformF	this study	*Chiloglottis formicifera*	NSW	Nattai NP	W	A
CHILgram01	this study	*Chiloglottis grammata*	TAS	Mt Wellington	W	E
CHILplur01	this study	*Chiloglottis pluricallata*	NSW	Barrington Tops NP	W	E
CHILplur02	this study	*Chiloglottis pluricallata*	NSW	Barrington Tops NP	W	E
CHILsemi01	Peakall et al. 2021	*Chiloglottis seminuda*	NSW	Blue Mountains NP	W	E
CHILsylv01	this study	*Chiloglottis sylvestris*	NSW	Morton NP	W	E
CHILtrap01	Peakall et al. 2021	*Chiloglottis trapeziformis*	ACT	Black Mt NR	W	E
CHILtrap02	this study	*Chiloglottis trapeziformis*	ACT	ANBG	W	E
CHILtrapF	this study	*Chiloglottis trapeziformis*	ACT	Black Mt NR	W	A
CHILtric01	this study	*Chiloglottis triceratops*	TAS	Mt Barrow	W	E
CHILtril01	this study	*Chiloglottis trilabra*	NSW	Tallaganda State Forest	W	E
CHILtrilF	this study	*Chiloglottis trilabra*	ACT	Black Mt NR	W	A
CHILturf01	this study	*Chiloglottis turfosa*	NSW	Kosciuszko NP	W	E
CHILvali01	this study	*Chiloglottis valida*	VIC	Kingslake NP	W	E

CHILvali02	Peakall et al. 2021	*Chiloglottis valida*	NSW	Kanagara Boyd NP	W	E
CHILvaliF	this study	*Chiloglottis valida*	NSW	Kosciuszko NP	W	A

TABLE S2. Putative anthocyanins and flavonols in floral extracts of *Chiloglottis* calli tissues. The positions of sugar and acyl group attachment to the anthocyanidins or flavonol aglycones were not determined, and glucosides cannot be distinguished from isomeric galactosides using our methodology. Especially informative MSMS transitions are listed in bold. Table reproduced from Wong et al. (2022) with slight modifications as the data presented there and in the present study were acquired at the same time, however, asterisks indicate putative flavonol glycosides unique to the Valida clade.

Putative anthocyanin/ flavonol glycoside	Putative aglycone	Peak RT (min)	[M]+	[M]+ MSMS transitions (relative intensity)	[M-2H]-, [M-2H+H2O]-
**Anthocyanin**					
Cyanidin glucoside (CG)	Cyanidin	7.12	449.1076	**287.0547 (100)**, 288.0580 (8.5), 71.7628 (0.9), 286.9883 (0.7), 69.7407 (0.3)	447.0931, 465.1034
Cyanidin malonylglucoside (CmG)	Cyanidin	8.93	535.1086	**287.0546 (100)**, 288.0579 (7.4), 71.7628 (0.9), 286.9883 (0.7), 258.0482 (0.06)	533.0936, 551.1042
Pelargonidin glucoside (PelG)	Pelargonidin	7.54	433.1131	**271.0596 (100)**, 272.0630 (10.3), 67.7641 (1.0), 270.9985 (0.6), 69.7845 (0.4)	431.0979, 449.1090
Peonidin glucoside (PeoG)	Peonidin	8.03	463.1233	**301.0702 (100),** 302.0735 (10.5), 75.2666 (0.9), 300.9990 (0.7), 286.0462 (0.4)	461.1088, 479.1197
Peonidin malonylglucoside (PeomG)	Peonidin	9.76	549.1234	**301.0701 (100),** 302.0734 (8.5), 75.2666 (0.9), 300.9991 (0.7), 286.0456 (0.4)	547.1096, 565.1200
**Flavonol glycoside**					
Quercetin diglucoside (QGG)	Quercetin	8.91	627.1541	**303.0492 (100),** 85.0287 (6.8), 304.0526 (5.9), 127.0388 (2.0), 97.0285 (1.8), **465.1016 (1.0)**	
Kaempferol diglucoside (KGG)	Kaempferol	8.95	611.1599	**287.0545 (100**), 85.0287 (10.4), 288.0579 (7.1), 97.0286 (2.8), 127.0388 (2.2), **449.1056 (2.0)**	

Quercetin malonyl diglucoside (QmGG)	Quercetin	9.56	713.1547	**303.0493 (100),** 127.0389 (8.7), 85.0287 (8.4), 304.0527 (8.2), 109.0285 (5.3), **465.1026 (2.4)**
Quercetin glucoside (QG)	Quercetin	10.39	465.1024	**303.0494 (100),** 85.0287 (10.7), 304.0527 (9.5), 97.0286 (2.8), 127.0389 (2.5),
Unknown Quercetin pentose (QP)	Quercetin	10.48	435.0925	**303.0496 (100),** 73.02881 (15.7), 304.0534 (10.1), 195.0285 (4.6), 57.0341 (4.8)
Quercetin malonylglucoside (QmG)	Quercetin	10.83	551.1034	**303.0496 (100),** 127.0390 (11.7), 85.0288 (11.6), 109.0286 (9.1), 304.0529 (7.8)
Kaempferol glucoside (KG)	Kaempferol	11.43	449.1076	**287.0546 (100),** 85.0288 (9.3), 288.0580 (6.9, 97.0286 (3.0), 127.0389 (2.5)
Isorhamnetin diglucoside (IRGG)	Isorhamnetin	11.81	641.1708	**317.0651 (100),** 318.0686 (6.2), 85.0287 (4.4), 97.0286 (1.7), 79.2659 (1.6), **479.1159 (1.2)**
Kaempferide diglucoside (KdeGG)	Kaempferide	11.84	625.1761	**301.0701 (100),** 302.0735 (7.6), 85.0287 (7.2), **463.1230 (2.6),** 97.0285 (2.0)
Kaempferol malonylglucoside (KmG)	Kaempferol	11.86	535.1075	**287.0546 (100),** 127.0390 (11.0), 85.0287 (9.5), 109.0285 (6.9), 159.0285 (4.5)
Isorhamnetin malonyl diglucoside (IRmGG)	Isorhamnetin	12.21	727.1708	**317.0649 (100**), 318.0683 (9.3), 85.0287 (6.6), 127.0389 (6.0), 109.0284 (4.1), **479.1175 (2.8)**
Isorhamnetin, unknown hexose-pentose glycone (IRHP) *	Isorhamnetin	12.31	611.1602	**317.0650 (100**), 318.0684 (8.2), 73.0288 (3.9), 57.0341 (1.8), **479.1168 (1.7)**
Kaempferide, unknown hexose-pentose glycone (KdeHP) *	Kaempferide	12.4	595.1654	**301.0703 (100**), 302.0736 (5.96), **463.1236 (3.5),** 115.0393 (7.61.5), 127.0388 (0.7)
Kaempferide malonyl diglucoside (KdemGG)	Kaempferide	12.32	711.1749	**301.0704 (100**), 85.0288 (8.2), 302.0736 (7.7), 127.0390 (7.6), 109.0285 (5.4), **463.1222 (4.6)**

Isorhamnetin glucoside (IRG)	Isorhamnetin	13.89	479.1182	**317.0650 (100)**, 318.0684 (10.2), 79.2653 (0.9), 316.9880 (0.8), 85.0287 (0.7)
Isorhamnetin malonylglucoside (IRmG)	Isorhamnetin	14.34	565.1182	**317.0649 (100)**, 127.0389 (10.4), 85.0287 (8.8), 318.0684 (7.6), 109.0285 (6.7)

TABLE S3. Putative anthocyanin/flavonol glycoside (A) biosynthesis and (B) modification - related pathway genes in five *Chiloglottis* with exemplar homologs in other plants. CHILtrap, *Chiloglottis trapeziformis*; CHILsemi, *Chiloglottis seminuda*; CHILturf, *Chiloglottis turfosa*; CHILaffv, *Chiloglottis aff. valida*; CHILvali, *Chiloglottis* valida.

Symbol	Gene name	CHILtrap	CHILsemi	CHILturf	CHILaffv	CHILvali
**A.**						
CHS	Chalcone synthase	CtrCHS1<br>CtrCHS2<br>CtrCHS3	CseCHS1<br>CseCHS2	CtuCHS1	CavCHS1	CvaCHS1
CHI	Chalcone isomerase	CtrCHI1	CseCHI1	CtuCHI1	CavCHI1	CvaCHI1
F3H	Flavone 3-hydroxylase	CtrF3H2<br>CtrF3H1	CseF3H1<br>CseF3H2	CtuF3H1	CavF3H1	CvaF3H1<br>CvaF3H2
F3pH	Flavonoid 3’-hydroxylase	CtrF3pH1<br>CtrF3pH2<br>CtrF3pH3<br>CtrF3pH4	CseF3pH1<br>CseF3pH2<br>CseF3pH3<br>CseF3pH4<br>CseF3pH5	CtuF3pH1<br>CtuF3pH2<br>CtuF3pH3<br>CtuF3pH4	CavF3pH1<br>CavF3pH2<br>CavF3pH3<br>CavF3pH4	CvaF3pH1<br>CvaF3pH2<br>CvaF3pH3<br>CvaF3pH4<br>CvaF3pH5
DFR	Dihydroflavonol reductase	CtrDFR1	CseDFR1	CtuDFR1	CavDFR1	CvaDFR1
LDOX	Leucoanthocyanidin dioxygenase	CtrLDOX2<br>CtrLDOX1	CseLDOX1<br>CseLDOX2	CtuLDOX1	CavLDOX1	CvaLDOX1
FLS	Flavonol synthase	CtrFLS1<br>CtrFLS2	CseFLS1	CtuFLS1	CavFLS1	CvaFLS1
**B.**						
UF3GT	UDP-glucose:flavonoid 3-O-glucosyltransferase e.g. *VvUFGT*, *AtUGT78D2*, *Ip3GT*	CtrUFGTa	CseUFGTa	CtuUFGTa	CavUFGTa	CvaUFGTa
MAT	Anthocyanin/Flavonoid malonyltransferase e.g. *Dv3MaT*, *At3AT1*, *At3AT2*	CtrMAT1<br>CtrMAT2<br>CtrMAT3<br>CtrMAT4	CseMAT1<br>CseMAT2<br>CseMAT3	CtuMAT1<br>CtuMAT2<br>CtuMAT3	CavMAT1<br>CavMAT2	CvaMAT1<br>CvaMAT2<br>CvaMAT3
5GT	Anthocyanin/Flavonoid 5-O-glucosyltransferase e.g. *Ih5GT*	Ctr5GT1<br>Ctr5GT2<br>Ctr5GT3	Cse5GT1<br>Cse5GT2	Ctu5GT1<br>Ctu5GT2	Cav5GT1<br>Cav5GT2	Cva5GT1<br>Cva5GT2
7GT	Anthocyanin/Flavonoid 7-O-glucosyltransferase e.g. *DgAA7GT*	Ctr7GT1<br>Ctr7GT2	Cse7GT1	Ctu7GT1	Cav7GT1	Cva7GT1
53GT	Anthocyanidin 5,3-O-glucosyltransferase e.g. *RhGT1*	Ctr53GT	Cse53GT	Ctu53GT	Cav53GT	Cva53GT
AOMT	Anthocyanin/Flavonoid O-methyltransferase e.g. *VvAOMT*, *VvFAOMT*, *PhMF2*, *PhMT2*	CtrFOMT2<br>CtrFOMT3<br>CtrFOMT1<br>CtrFOMT4	CseFOMT1<br>CseFOMT2<br>CseFOMT3	CtuFOMT1<br>CtuFOMT2	CavFOMT1<br>CavFOMT2	CvaFOMT1<br>CvaFOMT2<br>CvaFOMT3
